# Antidiabetic Activity of Differently Regioselective Chitosan Sulfates in Alloxan-Induced Diabetic Rats

**DOI:** 10.3390/md13053072

**Published:** 2015-05-15

**Authors:** Ronge Xing, Xiaofei He, Song Liu, Huahua Yu, Yukun Qin, Xiaolin Chen, Kecheng Li, Rongfeng Li, Pengcheng Li

**Affiliations:** 1 Institute of Oceanology, Chinese Academy of Sciences, Qingdao 266071, China; E-Mails: hexiaofei12@mails.ucas.ac.cn (X.H.); sliu@qdio.ac.cn (S.L.); yuhuahua@qdio.ac.cn (H.Y.); ykqin@qdio.ac.cn (Y.Q.); chenxl@qdio.ac.cn (X.C.); lkc@qdio.ac.cn (K.L.); rongfengli@qdio.ac.cn (R.L.); 2 College of Earth Science, University of the Chinese Academy of Sciences, Beijing 100049, China

**Keywords:** differently regioselective chitosan sulfates, hypoglycemic activity, glucose tolerance, plasma insulin, alloxan-induced diabetic rats

## Abstract

The present study investigated and compared the hypoglycemic activity of differently regioselective chitosan sulfates in alloxan-induced diabetic rats. Compared with the normal control rats, significantly higher blood glucose levels were observed in the alloxan-induced diabetic rats. The differently regioselective chitosan sulfates exhibited hypoglycemic activities at different doses and intervals, especially 3-*O*-sulfochitosan (3-S). The major results are as follows. First, 3,6-di-*O*-sulfochitosan and 3-*O*-sulfochitosan exhibited more significant hypoglycemic activities than 2-*N*-3, 6-di-*O*-sulfochitosan and 6-*O*-sulfochitosan. Moreover, 3-*S*-treated rats showed a more significant reduction of blood glucose levels than those treated by 3,6-di-*O*-sulfochitosan. These results indicated that –OSO_3_^−^ at the C3-position of chitosan is a key active site. Second, 3-S significantly reduced the blood glucose levels and regulated the glucose tolerance effect in the experimental rats. Third, treatment with 3-S significantly increased the plasma insulin levels in the experimental diabetic rats. A noticeable hypoglycemic activity of 3-S in the alloxan-induced diabetic rats was shown. Clinical trials are required in the future to confirm the utility of 3-S.

## 1. Introduction

Diabetes mellitus (DM) is a common group of metabolic diseases associated with endocrine and metabolic disorders, which are mainly characterized by hyperglycemia, with a genetic predisposition. DM leads to abnormal metabolism of carbohydrates, fats and proteins, sometimes accompanied by the long-term complications of diabetes, including microvascular, macrovascular, and neuropathic disorders [1]. DM affects human eyes, kidneys, hearts, nerves and blood vessels. According to previous reports, diabetes mellitus has become the third most serious threat to human health following malignant tumors and cardiovascular and cerebrovascular disease.

The latest statistical data of the International Diabetes Federation (IDF) showed that at least 382 million people worldwide had diabetes in 2013. Compared with 371 million cases in 2012, the increasing rate reached 8.4 percent, and by 2025, the organization predicts that there will be 592 million cases. Moreover, IDF showed that there are 5.1 million deaths caused by this disease per year, or one death every 6 seconds. The expense for the treatment of diabetes is high: The global diabetes medical costs are $548 billion, accounting for 11% of the global medical expenditure, and this is likely to rise to $627 billion by 2035. It has become a heavy economic burden of the individual, family and society. In China, 114 million people had diabetes in 2013, which means there is one Chinese patient for every three to four patients with diabetes mellitus in the world, and the amount of patients is expected to increase a few million per year. Therefore, research on the prevention and treatment of diabetes and its complications has become a major public health issue.

Currently available therapies for diabetes include insulin and various oral hypoglycemic agents, such as sulfonylureas, biguanides, metformin, glucosidase inhibitors, troglitazone, *etc.* [2]. In conventional therapy, insulin-dependent diabetes mellitus or type 1 is treated with exogenous insulin while the non-insulin-dependent diabetes mellitus or type 2 is treated with oral hypoglycemic agents [3,4]. However, these drugs have serious side effects. For example, sulfonylureas drugs may cause abnormal liver function and hypoglycemia and are also not recommended for pregnant women because of their teratogenic effects on the fetus. A large dose of biguanide drugs can lead to gastrointestinal reactions, including nausea, vomiting, abdominal pain, diarrhea, and loss of appetite. Patients with lung, liver, and kidney diseases are prone to lactic acidosis after taking biguanide drugs [5,6,7]. The other classes of antidiabetic drugs, such as insulin sensitizing agents, insulin antagonistic hormone inhibitors, gluconeogenesis inhibitors, insulin like growth factor, ISU (insulin) secretion, and traditional Chinese medicine preparations, including flavonoids, alkaloids and so on, are also not ideal. Therefore, the development of safer, more specific and more effective hypoglycemic agents is important for diabetes treatment.

A previous study found that chitin/chitosan has definite hypoglycemic effects. The presumed mechanism showed that chitosan of a certain molecular weight stimulated the beta-cells proliferation [8], the secretion and release of insulin, and limited the glucagon secretion of islet α cells. Moreover, chitosan was active on the liver: it inhibited hepatic gluconeogenesis and the *in vivo* absorption of sugar; reduced sugar output; enhanced the utilization of sugar by the surrounding tissue, thus reducing the level of blood sugar. Another hypothesis is that chitosan could increase the amounts of insulin and glucose receptors, improve insulin sensitivity, and strengthen the biological activity of the receptor. Subsequently, the intracellular oxidase system was inhibited followed by tissue hypoxia. As a result, glucose metabolism was increased, and the blood sugar decreased.

Some research has shown that differently regioselective sulfate chitosans had varying bioactivities. For example, the anticoagulant activity of 6-*O*-sulfochitosan (6-S) was notably higher than that of 2-*N*-sulfochitosan (2-S) and 3-*O*-sulfochitosan (3-S). However, the selective sulfation at N-2 and/or O-3 had a much higher inhibitory effect on the infection of the AIDS virus *in vitro* than that of the known 6-S [9]. Moreover, many studies showed that diabetes was associated with oxidative stress, which contributed to an increased production of reactive oxygen species (ROS), including superoxide radicals, hydroxyl radicals, lipid peroxidation, and hydrogen peroxide [10,11]. Antioxidants could thus be a potential type of drug for the treatment of diabetes [12]. Non-toxic and natural antioxidants have been shown to prevent oxidative damage in diabetes [13]. Our previous research showed that sulfate chitosans had obvious antioxidant activities [14]. Therefore, the present study investigated the anti-diabetic activity of differently regioselective sulfate chitosans in alloxan-induced diabetic rats. The results showed that 3-S markedly lowered the blood glucose level, improved the glucose tolerance of rats and increased the fasting serum insulin level of alloxan-induced diabetic rats. Based on our study, 3-S could potentially be developed as a new hypoglycemic drug.

## 2. Results

### 2.1. Physico-Chemical Parameter of Differently Regioselective Sulfate Chitosans

Table 1 shows the result of differently regioselective sulfate chitosans under the aforementioned reaction conditions. All products have good solubility.

**Table 1:** Characteristics of differently regioselective chitosan sulfates.

Species	Molecular Weight (×10^4^)	Sulfur Content (%)	Color of Resultant	Solubility
H2,3,6-S	12.4	14.7	Pale yellow	Easily soluble
3,6-S	11.7	12.1	White	Easily soluble
3-S	12.1	5.2	Yellow	Easily soluble
6-S	13.5	7.6	White	Soluble
L2,3,6-S	0.9	14.5	Pale yellow	Easily soluble
CTS	76	0	Pale yellow	Not soluble

### 2.2. Structural Characterization of All Chitosan Sulfates

**Figure 1:**
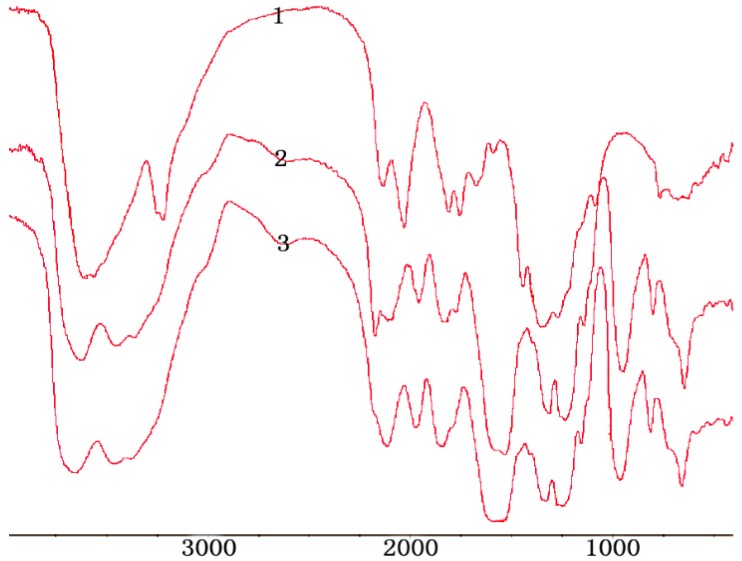
FTIR of H2,3,6-S (1) chitosan; (2) H2,3,6-S under dichloroacetic acid; (3) H2,3,6-S under formic acid.

In the FTIR (Fourier Transform Infrared) spectrum (as shown in Figure 1), characteristic absorptions at 1222 and 806 cm^−1^, due to sulfo groups, were assigned to S = O and C–O–S bond stretching, respectively.

**Figure 2:**
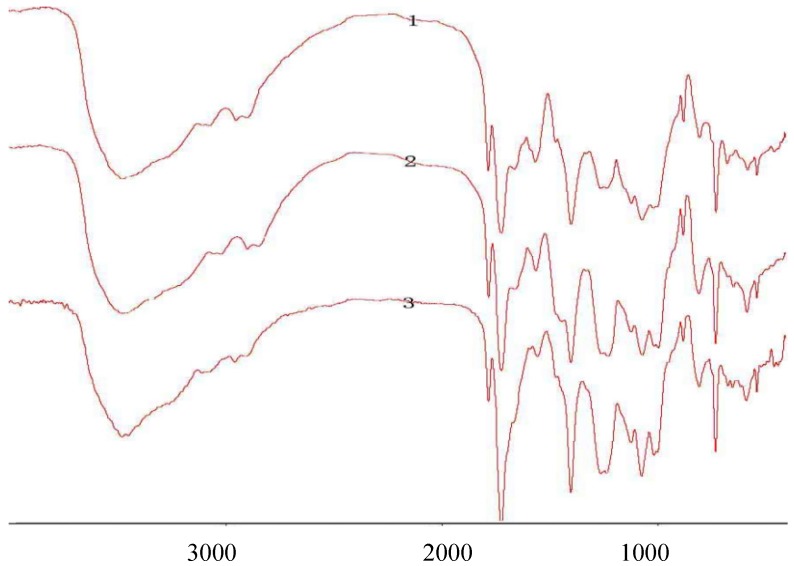
FTIR of 2-phthalimido-chitosan under 90 °C; 1: FTIR of 2-phthalimido-chitosan under 3.5 h; 2: FTIR of 2-phthalimido-chitosan under 3.0 h; 3: FTIR of 2-phthalimido-chitosan under 2.0 h.

**Figure 3:**
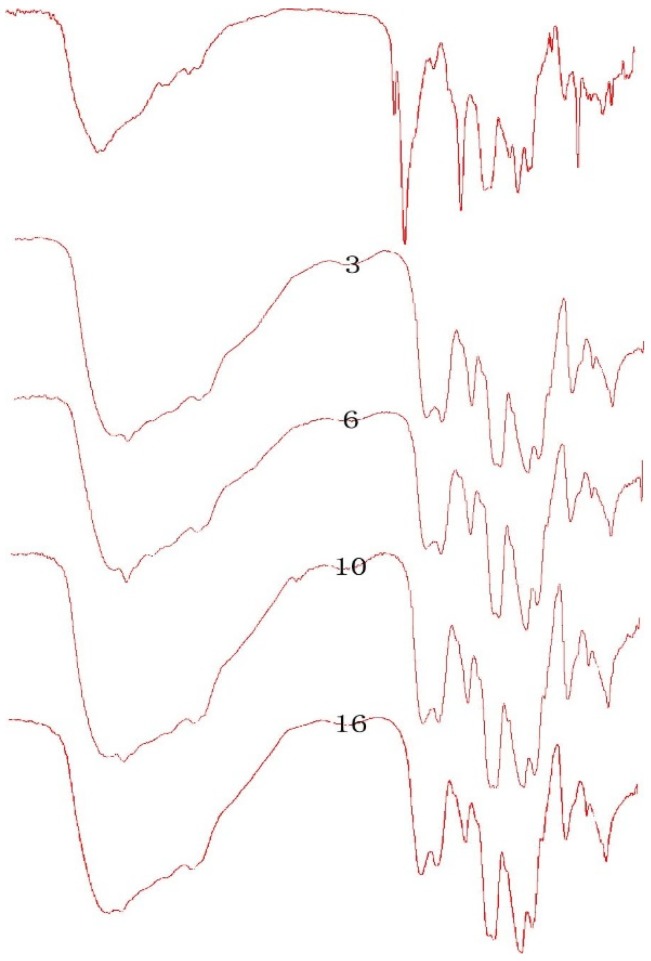
FTIR of 3,6-S; 3: Eliminating the phthalimido group under 3 h; 6: Eliminating the phthalimido group under 6 h; 10: Eliminating the phthalimido group under 10 h; 16: Eliminating the phthalimido group under 16 h.

**Figure 4:**
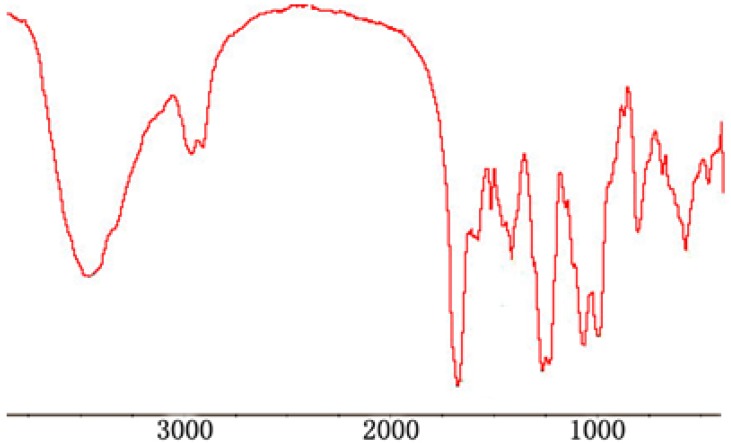
FTIR of 3-S.

The structures of 2-phthalimidochitosan, 3,6-S and 3-S were further investigated by means of FTIR spectrum (Figure 2, Figure 3 and Figure 4). In the FTIR spectrum (as shown in Figure 2), characteristic absorptions at 1712 cm^−1^ and 749 cm^−1^, due to phthalimido groups, were assigned to C=O and C–H bond stretching, respectively. Figure 3 shows that the phthalimido group was completely eliminated up to 3 h. As shown in Figure 4, the structure of 3-S was exhibited. Characteristic absorptions at 1261 and 805 cm^−1^ were assigned to S=O and C–O–S bond stretching, respectively. In this FTIR spectrum, three characteristic absorptions of the amino group, 1667.46, 1571.16, 1509.30 cm^−1^, appeared. Moreover, characteristic absorption of CH_2_ in C6 (2980 cm^−1^) was determined, which proved the 6-*O*-sulfo group was completely eliminated. Therefore, from the aforementioned result, 3,6-S and 3-S were successfully synthesized.

**Figure 5:**
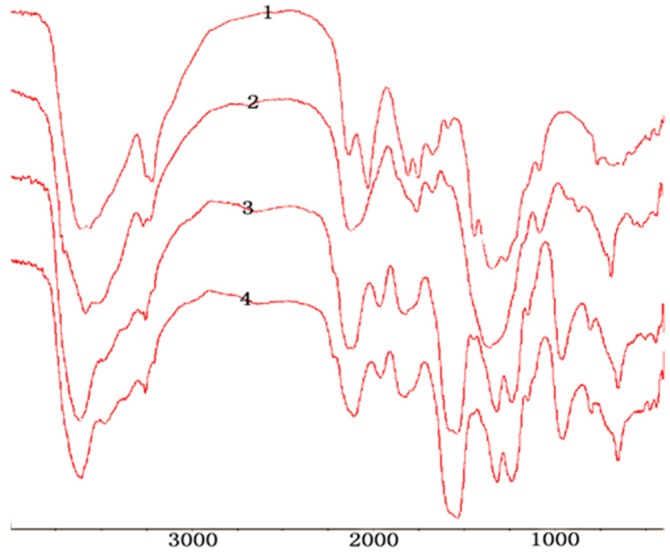
FTIR of 6-S; 1: Chitosan; 2: Cu-chitosan chelation; 3: Cu- sulfated chitoan chelation under formic acid; 4: Cu- sulfated chitoan chelation without formic acid.

The structures of 6-S was investigated by means of FTIR spectrum (Figure 5). In the FTIR spectrum, characteristic absorptions at 1225 cm^−1^ and 805 cm^−1^ were assigned to S=O and C–O–S bond stretching, respectively. Characteristic absorptions of hydroxy group 3440 cm^−1^ did not change in Cu-chitosan chelation and Cu-sulfated chitoan chelation, which proved that the C2-N-group and C3-O-group were completely protected, and a sulfated reaction did not destroy the protection group.

**Figure 6:**
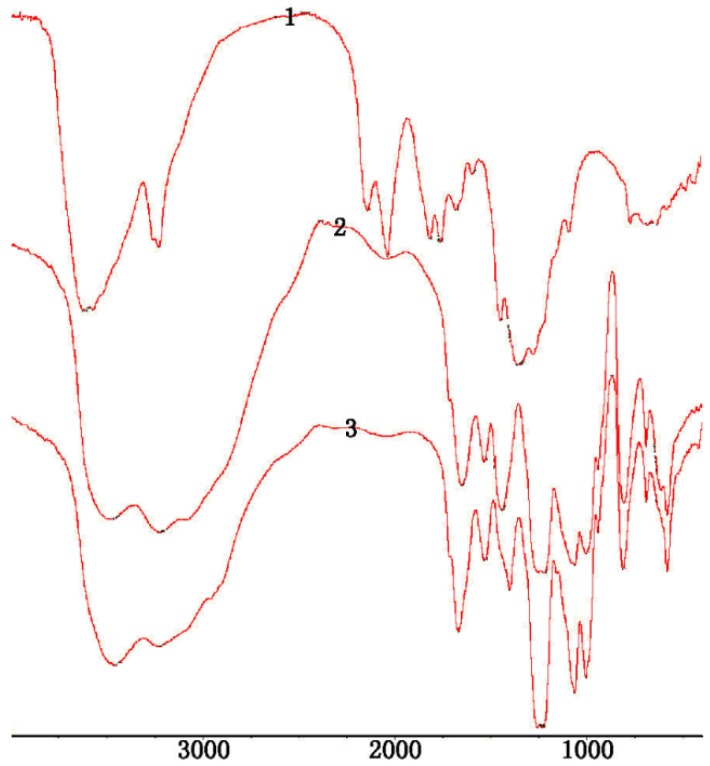
FTIR of L2,3,6-S; 1: Chitosan; 2: L2,3,6-S under traditional heating; 3: L2,3,6-S under microwave radiation (800W).

In the FTIR spectrum (as shown in Figure 6), characteristic absorptions at 1222 and 806 cm^−1^, due to sulfo groups, were assigned to S=O and C–O–S bond stretchings, respectively. The peak at 940 cm^−1^, due to the pyranose units in the polysaccharide, proved that the cyclic pyranosyl rings were not destroyed by microwave radiation.

### 2.3. The Effects of Differently Regioselective Sulfate Chitosans on Body Weight

As shown in Table 2, compared with the normal control group, the body weight of the diabetic model control group was significantly reduced (*p* < 0.05, *p* < 0.01, *p* < 0.01) on day 12, day 18 and day 30 after alloxan treatment. The body weight of the treatment groups did not significantly change compared to the normal control group. Moreover, the body weight of the 3-S treatment groups almost recovered to the normal level, especially the 150 mg/kg and 50 mg/kg dose groups. These results showed that the differently regioselective sulfate chitosan samples did not affect the rats’ body weights and had no negative effect on the rats.

### 2.4. Determination of Antidiabetic Activity of Differently Regioselective Chitosan Sulfates in Vivo

The effects of the differently regioselective sulfate chitosans on the fasting blood glucose levels of alloxan-induced diabetic rats are shown in Table 3. The administration of a single intraperitoneal injection of 50 mg/kg body weight of alloxan monohydrate induced diabetes in rats after 72 h. The fasting blood glucose levels in alloxan-induced diabetic rats were 21.83–27.01 mmol/L. The fasting blood glucose levels of the diabetic model rats were significantly higher than that of the normal control group. Differently regioselective sulfated chitosans have different hypoglycemic activities. Compared with the diabetic model rats, all doses of H2,3,6-S reduced the blood glucose levels of the rats tested on the 6th, 12th, 18th, 24th and 30th days to different degrees, although the differences were non-significant. Furthermore, we found that the hypoglycemic activity of low molecular weight L2,3,6-S is better than that of high molecular weight H2,3,6-S. A high dose of L2,3,6-S (400 mg/kg) showed a significant reduction (*p* < 0.05, *p* < 0.05) of the blood glucose level of the diabetic rats on the 12th and 18th days post-treatment. Hypoglycemic activities of sulfate chitosans depend on the substitution sites of the sulfate group. First, hypoglycemic activities of the 6-S groups are basically the same as that of the H2,3,6-S groups. Second, hydrazine hydrate could not be treated completely for 3,6-S, treatment with 3,6-S at a dose of 400 mg/kg and 150 mg/kg caused experimental animal mortality. However, treatment with 3,6-S at a low dose of 50 mg/kg in the diabetic rats led to a significant reduction (*p* < 0.05) in the blood glucose level on the 18th day. The results showed that the hypoglycemic activities of sulfate chitosans are enhanced by the introduction of sulfur at site 3. Third, all doses of the 3-S treatment reduced the blood glucose level in the diabetic rats significantly. Treatment with 3-S at a dose of 400 mg/kg caused a significant reduction (*p* < 0.05, *p* < 0.05, *p* < 0.05) in blood glucose levels in the diabetic rats on the 12th, 18th and 24th day post-treatment. Treatment with 3-S at a dose of 50 mg/kg led to a significant reduction (*p* < 0.01, *p* < 0.05) in blood glucose levels on the 12th and 18th day. The highest anti-hyperglycemic activity of 3-S was the 150 mg/kg dose in diabetic rats on the 12th, 18th, 24th and 30th day post-treatment (*p* < 0.01, *p* < 0.001, *p* < 0.001, *p* < 0.05). Therefore, these results indicated that 3-S had the highest hypoglycemic activity, and all of the investigated sulfate chitosans reduced the blood glucose levels in a dose-independent manner.

**Table 2:** The effects of differently regioselective chitosan sulfates on the body weights of alloxan-induced diabetic rats.

Group	Treatment	0th Day	6th Day	12th Day	18th Day	24th Day	30th Day
1	Normal control	183.9 ± 12.1 (10)	197.1 ± 13.6 (10)	219.0 ± 19.1 (10)	233.3 ± 23.0 (10)	237.9 ± 23.9 (10)	261.3 ± 40.3 (10)
2	Diabetic control (DC)	171.8 ± 25.8 (13)	178.3 ± 34.4 (12)	184.1 ± 39.8 (11) ^∆^	191.4 ± 37.1 (11) ^∆∆^	203.2 ± 47.9 (10)	201.8 ± 49.4 (10) ^∆∆^
3	DC + phenformin hydrochloride (100 mg/kg)	175.4 ± 31.4 (12)	181.1 ± 33.2 (10)	189.6 ± 35.0 (9)	199.0 ± 34.5 (9)	199.7 ± 41.8 (9)	208.1 ± 45.2 (8)
4	DC + H2,3,6-S (400 mg/kg)	174.0 ± 14.1 (9)	191.6 ± 11.0 (7)	178.9 ± 22.9 (7)	200.2 ± 21.4 (6)	209.8 ± 22.3 (6)	213.2 ± 20.9 (6)
5	DC + H2,3,6-S (150 mg/kg)	183.3 ± 25.7 (8)	200.4 ± 28.8 (7)	186.1 ± 23.9 (7)	207.4 ± 30.3 (7)	200.5 ± 29.5 (6)	207.7 ± 36.6 (6)
6	DC + H2,3,6-S (50 mg/kg)	185.9 ± 10.5 (8)	201.3 ± 15.1 (7)	198.4 ± 23.4 (7)	218.7 ± 30.6 (7)	224.4 ± 42.9 (7)	223.3 ± 44.5 (7)
7	DC + L2,3,6-S (400 mg/kg)	180.3 ± 14.6 (9)	198.8 ± 8.1 (8)	201.8 ± 11.1 (8)	207.5 ± 31.7 (8)	232.3 ± 27.5 (6)	234.3 ± 41.1 (6)
8	DC + L2,3,6-S (150 mg/kg)	170.8 ± 11.3 (8)	188.9 ± 18.2 (7)	187.9 ± 25.3 (7)	199.0 ± 32.5 (7)	213.1 ± 46.8 (7)	205.9 ± 48.5 (7)
9	DC + L2,3,6-S (50 mg/kg)	178.1 ± 23.7 (9)	200.9 ± 26.0 (7)	204.9 ± 28.3 (7)	217.1 ± 37.4 (7)	228.4 ± 49.1 (7)	228.1 ± 38.8 (7)
10	DC + 6-S (400 mg/kg)	173.4 ± 18.1 (9)	189.5 ± 23.7 (8)	202.4 ± 24.1 (7)	213.0 ± 31.7 (7)	199.0 ± 21.7 (6)	201.6 ± 20.5 (5)
11	DC + 6-S (150 mg/kg)	183.3 ± 13.9 (9)	195.3 ± 18.4 (8)	206.5 ± 23.1 (8)	222.5 ± 36.9 (8)	240.1 ± 43.7 (8)	238.4 ± 41.0 (8)
12	DC + 6-S (50 mg/kg)	179.6 ± 23.1 (8)	189.4 ± 33.9 (8)	196.5 ± 42.4 (8)	201.9 ± 50.1 (8)	222.9 ± 63.6 (8)	223.4 ± 72.9 (8)
13	DC + 3,6-S (400 mg/kg)	170.2 ± 20.1 (9)	±	±	±	±	±
14	DC + 3,6-S (150 mg/kg)	168.8 ± 6.11 (9)	±	±	±	±	±
15	DC + 3,6-S (50 mg/kg)	167.7 ± 15.2 (7)	175.9 ± 16.3 (7)	188.0 ± 29.4 (7)	193.1 ± 34.8 (7)	196.6 ± 46.9 (7)	211.7 ± 47.3 (6)
16	DC + 3-S (400 mg/kg)	173.8 ± 25.0 (9)	195.9 ± 32.1 (8)	200.5 ± 35.1 (8)	215.5 ± 47.4 (8)	221.0 ± 63.5 (8)	221.9 ± 65.8 (8)
17	DC + 3-S (150 mg/kg)	176.0 ± 28.5 (9)	192.5 ± 41.0 (8)	208.4 ± 38.6 (7)	232.7 ± 42.7 (6)	243.5 ± 48.8 (6)	244.2 ± 59.9 (6)
18	DC + 3-S (50 mg/kg)	189.9 ± 27.1 (8)	207.6 ± 38.3 (7)	221.2 ± 46.4 (6)	230.8 ± 39.2 (6)	245.8 ± 50.8 (6)	247.2 ± 51.9 (6)
19	DC + CTS (400 mg/kg)	183.0 ± 21.2 (9)	200.6 ± 31.7 (7)	207.5 ± 44.6 (6)	214.2 ± 50.4 (5)	221.0 ± 65.9 (5)	236.5 ± 62.7 (4)
20	DC + CTS (150 mg/kg)	174.7 ± 16.4 (9)	188.1 ± 22.2 (8)	201.0 ± 31.5 (7)	215.1 ± 32.5 (7)	224.6 ± 44.7 (7)	219.0 ± 46.9 (7)
21	DC + CTS (50 mg/kg)	184.1 ± 23.6 (9)	197.4 ± 31.1 (7)	194.7 ± 37.6 (7)	198.1 ± 42.9 (7)	203.8 ± 29.7 (5)	212.5 ± 43.2 (4)

Readings are values ± S.E.; (*n*) = number of animals in each group; ^∆^ *p* < 0.05, ^∆∆^ *p* < 0.01 *vs.* normal control.

**Table 3:** The effects of differently regioselective chitosan sulfates on the fasting blood glucose level of alloxan-induced diabetic rats.

Group	Treatment	0th Day	6th Day	12th Day	18th Day	24th Day	30th Day
1	Normal control	4.94 ± 0.64 (10)	4.96 ± 0.39 (10)	4.97 ± 0.37 (10)	5.84 ± 0.89 (10)	5.74 ± 1.13 (10)	5.70 ± 1.06 (10)
2	Diabetic control (DC)	23.02 ± 6.77 (13) ^∆∆∆^	24.22 ± 7.97 (12) ^∆∆∆^	26.59 ± 6.77 (11) ^∆∆∆^	27.01 ± 7.49 (11) ^∆∆∆^	24.06 ± 4.37 (10) ^∆∆∆^	21.83 ± 6.66(10) ^∆∆∆^
3	DC + phenformin hydrochloride (100 mg/kg)	24.08 ± 5.87 (12)	20.82 ± 7.69 (10)	17.16 ± 7.27 (9) **	14.73 ± 6.23 (9) ***	13.48 ± 4.45 (9) ***	14.84 ± 6.09 (8) *
4	DC + H2,3,6-S (400 mg/kg)	22.60 ± 6.82 (9)	22.84 ± 6.23 (7)	19.74 ± 7.13 (7)	23.88 ± 7.55 (6)	22.12 ± 7.79 (6)	18.12 ± 8.29 (6)
5	DC + H2,3,6-S (150 mg/kg)	23.39 ± 6.96 (8)	24.89 ± 6.11 (7)	23.54 ± 7.03 (7)	27.34 ± 3.79 (7)	21.02 ± 5.22 (6)	22.03 ± 6.78 (6)
6	DC + H2,3,6-S (50 mg/kg)	22.49 ± 6.06 (8)	19.96 ± 7.65 (7)	20.64 ± 8.68 (7)	18.39 ± 10.86 (7)	16.16 ± 10.16 (7)	17.23 ± 9.39 (7)
7	DC + L2,3,6-S (400 mg/kg)	23.23 ± 6.57 (9)	21.41 ± 2.29 (8)	20.28 ± 2.88 (8) *	17.68 ± 6.93 (8) *	15.85 ± 10.59 (6)	15.68 ± 8.39 (6)
8	DC + L2,3,6-S (150 mg/kg)	22.76 ± 6.42 (8)	20.99 ± 7.24 (7)	21.14 ± 6.11 (7)	22.40 ± 6.15 (7)	22.56 ± 5.14 (7)	23.80 ± 6.49 (7)
9	DC + L2,3,6-S (50 mg/kg)	24.11 ± 7.58 (9)	23.76 ± 3.17 (7)	20.56 ± 6.73 (7)	19.50 ± 8.274 (7)	20.26 ± 8.91 (7)	20.73 ± 9.69 (7)
10	DC + 6-S (400 mg/kg)	22.26 ± 5.89 (9)	27.11 ± 7.62 (8)	21.57 ± 8.26 (7)	22.46 ± 10.55 (7)	17.52 ± 9.25 (6)	23.50 ± 10.82 (5)
11	DC + 6-S (150 mg/kg)	22.06 ± 6.88 (9)	20.33 ± 7.93 (8)	20.40 ± 7.06 (8)	18.43 ± 9.35 (8)	20.40 ± 9.53 (8)	16.51 ± 9.60 (8)
12	DC + 6-S (50 mg/kg)	22.98 ± 7.23 (8)	26.98 ± 4.90 (8)	19.99 ± 8.28 (8)	19.53 ± 10.44 (8)	19.64 ± 11.57 (8)	16.58 ± 9.22 (8)
13	DC + 3,6-S (400 mg/kg)	22.59 ± 5.98 (9)	±	±	±	±	±
14	DC + 3,6-S (150 mg/kg)	24.19 ± 7.66 (9)	±	±	±	±	±
15	DC + 3,6-S (50 mg/kg)	26.14 ± 6.50 (7)	22.77 ± 8.32 (7)	20.41 ± 6.25 (7)	19.89 ± 5.27 (7) *	19.73 ± 8.97 (7)	22.08 ± 8.89 (6)
16	DC + 3-S (400 mg/kg)	24.09 ± 7.55 (9)	21.91 ± 9.62 (8)	19.21 ± 7.75 (8) *	20.31 ± 8.96 (8) *	19.50 ± 8.14 (8) *	22.08 ± 8.89 (8)
17	DC + 3-S (150 mg/kg)	23.79 ± 7.77 (9)	22.90 ± 5.10 (8)	18.63 ± 5.22 (7) **	18.22 ± 3.41 (6) **	17.55 ± 3.29 (6) **	16.13 ± 4.36 (6) *
18	DC + 3-S (50 mg/kg)	22.88 ± 7.85 (8)	22.84 ± 6.15 (7)	17.65 ± 4.20 (6) **	18.45 ± 5.14 (6) *	22.43 ± 5.86 (6)	18.70 ± 6.32 (6)
19	DC + CTS (400 mg/kg)	23.94 ± 8.06 (9)	21.37 ± 5.44 (7)	19.75 ± 5.48 (6) *	21.92 ± 7.72 (5)	21.74 ± 9.90 (5)	23.20 ± 8.00 (4)
20	DC + CTS (150 mg/kg)	22.88 ± 7.41 (9)	21.98 ± 7.52 (8)	19.74 ± 6.87 (7)	21.13 ± 9.53 (7)	18.49 ± 8.51 (7)	18.86 ± 9.72 (7)
21	DC + CTS (50 mg/kg)	24.21 ± 8.24 (9)	23.83 ± 7.90 (7)	21.77 ± 7.91 (7)	24.00 ± 8.78 (7)	22.68 ± 9.09 (5)	23.83 ± 4.35 (4)

Readings are values ± S.E.; (*n*) = number of animals in each group; ^∆∆∆^ *p* < 0.001 *vs.* normal control; * *p* < 0.05; ** *p* < 0.01; *** *p* < 0.001 *vs.* diabetic control.

### 2.5. Effect of 3-S on the Sugar Tolerance of Normal Rats

As described above, 3-S had the highest hypoglycemic activity among all of the selected sulfate chitosans. Therefore, 3-S was further investigated for its activity of increasing the sugar tolerance of the alloxan-induced rats.

**Figure 7:**
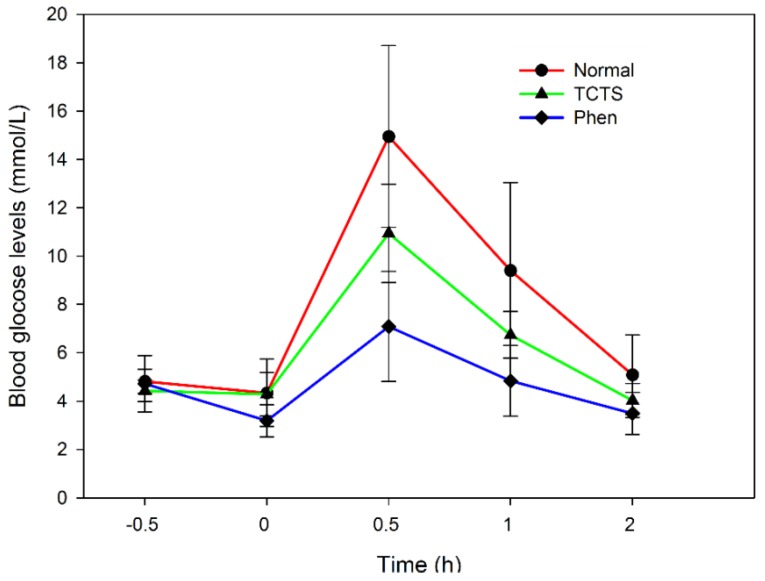
Hypoglycemic effects of 3-S on the fasting blood glucose levels of normal rats during GTT, each value shown in mean ± S.E.; *n* = 10, number of animals in each group.

Table 4 and Figure 7 showed the fasting blood glucose levels of normal control, and 3-S- and phenformin hydrochloride-treated rats after intraperitoneal administration of glucose (2 g/kg body weight). As shown in Table 4 and Figure 7, in the three groups, the blood glucose concentration peaked 0.5 h after the intraperitoneal administration of glucose. However, compared with the normal control group, the groups treated with 3-S (300 mg/kg) and phenformin hydrochloride (200 mg/kg) exhibited significantly lower blood glucose levels (*p* < 0.01, *p* < 0.001, respectively). After 0.5 h, the blood glucose levels of all of the experimental rats decreased. Moreover, 3-S- and phenformin hydrochloride-treated rats had significantly lower blood glucose concentrations at 1 and 2 h compared to the normal control rats.

**Table 4:** Glucose tolerance tests in normal and experimental groups.

Group	Dose (mg/kg)	*n*	Prior to Treatment	After Treatment			
				0 h	0.5 h	1 h	2 h
Normal control		10	4.82 ± 0.50	4.34 ± 1.39	14.95 ± 3.76	9.41 ± 3.63	5.09 ± 1.64
3-S	300	10	4.43 ± 0.44	4.29 ± 0.90	10.94 ± 2.04 **	6.74 ± 0.97 *	4.03 ± 0.70 *
Phenformin hydrochloride	200	10	4.72 ± 1.17	3.19 ± 0.67 *	7.09 ± 2.28 ***	4.84 ± 1.46 **	3.49 ± 0.87 *

* *p* < 0.05; ** *p* *<* 0.01 *vs.* normal control.

### 2.6. The Effect of 3-S on Fasting Blood Glucose and Insulin Levels

Table 5 showed the levels of the fasting blood glucose and plasma insulin in normal control, diabetic control and experimental groups after 14 days of treatment. Compared with the normal control rats, the blood glucose level of the diabetic control rats was significantly increased, whereas the level of plasma insulin was significantly decreased. Treatment with 3-S at doses of 300 and 800 mg/kg caused a significant reduction in blood glucose level and a significant increase in serum insulin level. Moreover, the hypoglycemic activity of 3-S was higher than that of glibenclamide.

**Table 5:** The effect of 3-S on fasting serum insulin levels of normal and alloxan-induced diabetic rats.

Group	Treatment	*n*	Blood Glucose Level (mmol/L)		Fasting Serum Insulin Levels µIU/mL
			Before Treatment	After Treatment	
1	Normal control	10	5.17 ± 1.05	5.00 ± 0.81	6.71 ± 1.70
2	Diabetic control (DC)	10	32.10 ± 1.76 ^∆∆∆^	26.18 ± 5.68 ^∆∆∆^	3.54 ± 1.93 ^∆∆^
3	DC + 3-S (800 mg/kg)	10	30.25 ± 5.30	19.76 ± 9.20 *	5.44 ± 1.65 *
4	DC + 3-S (300 mg/kg)	10	29.96 ± 4.94	17.86 ± 7.93 **	5.12 ± 1.50 *
5	DC + 3-S (100 mg/kg)	10	31.78 ± 3.07	20.05 ± 7.28	4.00 ± 1.65
6	DC + Glibenclamide (25 mg/kg)	10	31.92 ± 2.63	26.45 ± 7.00	4.94 ± 1.85

Readings are values ± S.E.; (*n*) = number of animals in each group; ^∆∆^ *p* < 0.01; ^∆∆∆^ *p* < 0.001 *vs.* normal control; * *p* < 0.05; ** *p* < 0.01 *vs.* diabetic control.

## 3. Discussion

Type I diabetes is an autoimmune disease. Type I diabetics need insulin injections to survive, which sometimes cause a series of complications. The development of safer, more specific and more effective hypoglycemic agents are important. Therefore, this study is the preliminary assessment and comparison of the anti-diabetic activities of differently regioselective chitosan sulfates. The diabetic model was developed by the intraperitoneal injection of alloxan.

Alloxan, a hydrophilic and chemically unstable pyrimidine derivative, is one of the common substances administered to induce diabetes mellitus. Alloxan has a destructive effect on the pancreatic β cells because it can generate a massive amount of oxygen radicals [15,16]. Some studies have shown that free radicals can rapidly accumulate and lead to oxidative stress in diabetic animals, which might impair the function of the liver and kidney, decrease antioxidase activities and increase lipid peroxidation levels [17]. Therefore, the role of oxidative stress/antioxidant balance in diabetes and its complications is an important research topic. Much attention has been focused on the research of antioxidant substances. Based on our previous research, differently regioselective chitosan sulfates have metal chelating and free radical scavenging properties [14]. Therefore, we hypothesized that differently regioselective chitosan sulfates may have hypoglycemic activities.

In the present study, we found that the hypoglycemic activities of regioselective chitosan sulfates are related to the position of their substitute group, though all of the selected sulfated chitosans had hypoglycemic activities. However, substitution degree of sulfate is not a major factor for the effect of hypoglycemic activity *in vivo*. High molecular weight 2-*N*-3,6-di-*O*-sulfo chitosan (H2,3,6-S) and 6-*O*-sulfochitosan (6-S) have equal hypoglycemic activities that were weaker than that of 3,6-si-*O*-sulfochitosan (3,6-S) and 3-*O*-sulfochitosan (3-S). Among all of the investigated sulfated chitosans, 3-S has the highest hypoglycemic activity. Treatment with 3-S contributed to a significant reduction of the blood glucose levels in the alloxan-induced diabetic rats. The results indicated that –OSO_3_^−^ at the C3 position is important, as introduction of this substitute group significantly increased the hypoglycemic activity of chitosan. In addition, we found that the hypoglycemic activities of low molecular weight 2-*N*-3,6-di-*O*-sulfo chitosan (L2,3,6-S) were obviously higher than that of H2,3,6-S. Therefore, the molecular weight is another important factor influencing hypoglycemic activities of sulfated chitosans. In this study, the hypoglycemic activities of differently regioselective sulfate chitosans are consistent with their antioxidant activity. The ability of 3,6-S and 3-S to scavenge and chelate hydroxyl radicals and their reducing power were stronger than that of H2,3,6-S and 6-S. The antioxidant activity of L2,3,6-S was significantly higher than that of H2,3,6-S. It is noteworthy that the relationship between the dose and the hypoglycemic activities of all of the investigated sulfate chitosans showed a bell-shaped curve. For example, the dose of 3-S (150 mg/kg) exhibited the highest hypoglycemic effect. Its effect was more significant than that of the low (50 mg/kg) or high dose (400 mg/kg) of 3-S. This result suggested that the 150 mg/kg dose may be the effective hypoglycemic dose of 3-S. This result provided a theoretical basis for the pharmacological structure-function relationship among different backbone structures and differently arranged functional groups.

Glucose tolerance is the human tolerance to glucose. Clinical tests usually measure the glucose tolerance of patients suspected of having diabetes. After oral administration of glucose for 2 h, the body reduces the tolerance to glucose if the blood glucose levels range from 7.8 to 11.1 mmol/L. In other words, the sugar uptake and usage of the body are worse than normal. In the present study, 3-S lowered the blood glucose levels and regulated the glucose tolerance effect in experimentally induced rats. 3-S was able to enhance glucose utilization because it significantly decreased the blood glucose level in glucose-loaded rats. This effect may be due to the restoration of a delayed insulin response or the inhibition of the intestinal absorption of glucose. Lazarow *et al.*, [18] and Colca *et al.*, [19] described the mechanism of action of alloxan. According to their studies, alloxan caused a massive reduction in insulin release through the destruction of β cells of the islets of Langerhans.

The pancreas is the primary organ involved in sensing the organism’s dietary and energetic states via the glucose concentration in the blood; in response to elevated blood glucose, insulin is secreted [20]. When there are not enough available β cells to supply sufficient insulin to meet the needs of the body, insulin-dependent diabetes occurs [21]. In our study, as shown in Table 5, we observed a significant increase in the plasma insulin level when alloxan diabetic rats were treated with 3-S. At the same time, 3-S, at a dose of 300 mg/kg and 800 mg/kg body weight, was found to have a significant hypoglycemic activity and be more effective than glibenclamide (25 mg/kg). At a dose of 25 mg/kg, glibenclamide did not exhibit any hypoglycemic activity and only slightly increased fasting serum insulin levels. Therefore, the hypoglycemic potential of 3-S may be due to its ability to promote the renewal of β cells in the pancreas, help recover partially destroyed β cells, or stimulate pancreatic insulin secretion. However, the exact mechanism by which 3-S lowered the blood glucose level is not yet clear and needs to be further studied.

## 4. Materials and Methods

### 4.1. Materials and Chemicals

Alloxan and the reagents for serum insulin were purchased from Sigma-Aldrich Chemicals Co. (Saint Louis, MS, USA). A glucose analyzer and strips were purchased from Arkray Factory Inc. (Shiga, Japan). Phenformin hydrochloride tablets were purchased from Zhejiang Yatai Pharmaceutical Co. Ltd. (Shaoxing, Zhejiang, China). Glibenclamide tablets were purchased from Tianjin Lisheng Pharmaceutical Co. Ltd. (Tianjin, China). All other chemicals and reagents, unless otherwise specified, were not purified, dried or pretreated.

### 4.2. Experiment

#### 4.2.1. Preparation of Sulfating Reagent

Five milliliters of HClSO3 were added dropwise and stirred into 30 mL *N*,*N*-dimethylformamide (DMF) which was previously cooled at 0–4 °C. The reaction mixture was stirred without cooling until the solution (DMF·SO_3_) reached room temperature.

#### 4.2.2. The Preparation of Sulfated Chitosan of C2,3,6 Sulfation (H2,3,6-S)

Fifty milliliters of DMF·SO_3_ was added a 500 mL threenecked bottomed flask containing 50 mL of chitosan solution in a mixture of DMF–DCAA or DMF–formic acid with swirling to get gelatinous chitosan. Then the reaction was run at adequate temperature (40–60 °C) for 1–2.5 h, and 95% of EtOH (300 mL) was added to precipitate the product, giving a white precipitate. The mixture of products was filtered. The precipitate was washed with EtOH, then dissolved in distilled water, and the pH was adjusted to pH 7–8 with 2 M NaOH. The solution was dialyzed against distilled water for 48 h using an 8000 Da MW cut-off dialysis membrane. The product was then concentrated and lyophilized to give chitosan sulfate (2 g chitosan gave 2–3.7 g chitosan sulfates according to different conditions, including time, temperature and acid solvent).

#### 4.2.3. The Preparation of Sulfated Chitosan of C2,3,6 Sulfation (L2,3,6-S)

DMF·SO_3_ reagent (50 mL) was added to a 300 mL Erlenmeyer flask containing 50 mL of chitosan solution in a mixture of DMF–formic acid with swirling to get gelatinous chitosan. The Erlenmeyer flask containing the mixture of reactant was placed on the center of the turntable of the microwave oven. To control the reaction temperature to ~100 °C, another 50 mL Erlenmeyer flask containing a higher boiling solvent was also placed on the turntable in the microwave oven. Different irradiation powers and radiation times were set. After irradiation ceased, the reaction liquid was immediately poured into 90% EtOH (300 mL), giving a white precipitate. The mixture of products was filtered. The precipitate was washed with EtOH, then dissolved in distilled water, and the pH was adjusted to pH 7–8 with 2 M NaOH. The solution was dialyzed against distilled water for 48 h using a 3600 Da MW cutoff dialysis membrane. The product was then concentrated and lyophilized to give chitosan sulfate (2 g chitosan gave 1.8–3.1 g chitosan sulfated according to different conditions, including radiation power, radiation time, *etc.*).

#### 4.2.4. The Preparation of Sulfated Chitosan of C3,6 Sulfation (3,6-S)

An amount of 4 g of chitosan was suspended in 100 mL dry DMF and stirred, then 5 g phthalic anhydride and 3 mL ethylene glycol was added in this system by stirring. The mixture was stirred for 2 h at 90 °C, and the transparent yellow solution was poured into ice-cold water. The precipitate was filtered off, washed with water, resuspended in EtOH, filtered off again. The product was dried at 60 °C and obtained the 2-phthalimidochitosan. Then 50 mL DMF·SO3 was added dropwise to a 250 mL three-necked bottomed flask containing 2 g 2-phthalimidochitosan and 100 mL DMF. Then the reaction was run at 50 °C for 2 h, and 95% of EtOH (500 mL) was added to precipitate the product, giving a pale yellow precipitate. The mixture of products was filtered and washed with EtOH, then redissolved in distilled water, and the pH was adjusted to 7–8 with 2 M NaOH. The solution was dialyzed against distilled water for 48 h using a 3600 Da MW cut-off dialysis membrane. The product was then concentrated and lyophilized to give pale yellow 3,6-di-*O*-2-*N*-phthalimido-sulfochitosan.

3,6-di-*O*-2-*N*-Phthalimido-sulfochitosan was dissolved in deionized water, and hydrazine hydrate was added. The solution was heated to 70 °C for 3 h. Afterwards, water was added, and the solution evaporated nearly to dryness. This was repeated three to five times to eliminate the remaining hydrazine. Then, the solution was dialyzed against distilled water for 48 h using a 3600 Da MW cut-off dialysis membrane. The product was then concentrated and lyophilized to give pale yellow 3,6-di-*O*-sulfochitosan.

#### 4.2.5. The Preparation of Sulfated Chitosan of C3 Sulfation (3-S)

3,6-di-*O*-Sulfochitosan was dissolved in deionized water, and then the mixture of *N*-methylpyrrolidinone and water was added to the aforementioned solution. The yellow solution was stirred for 3 h at 90 °C. After the reaction, the pH was adjusted to 9.0 by 2 M NaOH. The solution was dialyzed, concentrated and freeze-dried to give yellow 3-*O*-sulfochitosan (TCTS).

#### 4.2.6. The Preparation of Sulfated Chitosan of C6 Sulfation (6-S)

Chitosan was dissolved in 2% formic acid (50 mL), then 1 M CuSO_4_·5 H_2_O was added dropwise to the above-mentioned solution at rt. After stirring for 4 h, the PH was adjusted to 6–7 by 2% NH_3_·H_2_O, then the reaction was run at rt for 4 h. The resulting precipitate was filtered and washed with water, acetone, and Et_2_O. This precipitate was dispersed in dry DMF (30 mL) for 16 h, then SO_3_·DMF was added dropwise to the aforementioned mixture solution, and the reaction was run for 1–3 h at 40–60 °C. After the reaction, the pH was adjusted to 8 by saturated NaHCO_3_. The solution was dialyzed for 3 days. To eliminate the copper protective group from the complex, the aforementioned solution was passed through an Amberlite IRC 718 column. The eluate was neutralized and then freeze-dried.

#### 4.2.7. Analytical Methods

The degree of deacetylation of chitosan was 87% by potentionmetry and the viscosity average-molecular weight was 7.6 × 10^5^. FTIR spectra was measured by Nicolet Magna-Avatar 360 (American) with KBr disk; Sulfate content % was measured in a SC-132 sulfur meter (LECO), and the average viscometric molecular weight of sulfated chitosan was estimated from the intrinsic viscosity determined in the solvent 0.1 M CH_3_COOH/0.2 M NaCl using the Mark-Houwink parameters α = 0.96, Kη = 1.424 at 25 °C when the intrinsic viscosity was expressed in mL·g^−1^.

### 4.3. Animals

Wistar rats, due to their high fecundity, litter size, gentle temperament, strong resistance to infectious diseases, and low incidence of spontaneous tumors, are widely used in various fields of biomedical experimentation. Wistar rats of approximately the same age and a body weight of 180–220 g, half male and half female, were obtained from Tianjin Institute of Pharmaceutical Research and were used after being acclimatized to laboratory conditions for a week. The rats were provided a standard rat pellet diet and water. There were 21 groupsemployed, and each consisted of 5 to 10 animals. The rats were housed in stainless steel cages to provide them with sufficient space and to avoid unnecessary morbidity and mortality. All experimental procedures were performed in strict accordance with the recommendations in the Guide for the Care and Use of Laboratory Animals of the Institutional Animal Ethical Committee, and the protocols were approved by the Committee on the Ethics of Animal Experiments of the Institute of Oceanology, Chinese Academy of Sciences, Shandong, China. All efforts were made to minimize suffering, and the experimental animals were anesthetized using sodium pentobarbital before blood sampling was performed. The animals for the following experiments were pre-fasted overnight, but were allowed free access to water.

### 4.4. Studies on Alloxan-Induced Diabetic Rats

#### 4.4.1. Induction of Diabetes Mellitus

Diabetes was induced by the intraperitoneal injection of alloxan monohydrate in normal saline to overnight-fasted animals at a dose of 50 mg/kg body weight. After 72 h, the rats were deprived of food for 3 h, and then the blood glucose level was determined. The rats with blood glucose levels above 10 mmol/L were used for the study.

#### 4.4.2. Determination of the Hypoglycemic Effect on Diabetic Rats

Alloxan diabetic rats were divided into 23 groups of 5–10 animals each. Group 1 was the normal group, and Group 2 was the diabetic model control group. The groups were given an equivalent volume of saline (0.5 mL/100 g day body weight) by intragastric administration. For the Group 3 animals, phenformin hydrochloride was applied at a dose of 100 mg/kg body weight/day by intragastric administration. For the Group 4, Group 5 and Group 6 animals, sulfated chitosan of C_2,3,6_ sulfation (H2,3,6-S) was given at a dose of 50, 150 and 400 mg/kg body weight/day by intragastric administration. Group 7, Group 8 and Group 9 animals were treated with low molecular weight sulfated chitosan of C_2,3,6_ sulfation (L2,3,6-S). Group 10, Group 11 and Group 12 were treated with sulfated chitosan of C_6_ sulfation (6-S). Group 13, Group 14 and Group 15 were given sulfated chitosan of C_3,6_ sulfation (3,6-S). Group 16, Group 17 and Group 18 were given sulfated chitosan of C_3_ sulfation (3-S). Group 19, Group 20 and Group 21 were given chitosan (CTS). The groups were given equivalent doses of H2,3,6-S by intragastric administration, once a day for 30 days. The fasting blood samples (1 mL per rat) were collected on day 6, 12, 18, 24 and 30 to determine the glucose level.

#### 4.4.3. Glucose Tolerance Test

Kunming rats are an outbred rats group. China has the largest production and usage of this rat. After years of breeding, Kunming rats now have a very low rate of spontaneous tumors, strong resistance to disease and resilience, high reproductive rate and survival rate. Therefore, Kunming rats are widely used in various fields of biomedical experiments and account for approximately 70% of the total amount of all the rats. Kunming rats of approximately the same age and with a body weight of 20–30 g, half male and half female, were obtained from the Tianjin Institute of Pharmaceutical Research and were used after being acclimatized to laboratory conditions for a week. They were provided a standard rat pellet diet and water. Three groups were employed, and each consisted of 10 animals. Group 1 was the normal group that received an equivalent volume of saline. For the animals in Group 2, phenformin hydrochloride was administered at a dose of 200 mg/kg body weight/day and in Group 3 animals, 3-S was administered at a dose of 300 mg/kg body weight/day for 14 days. Fifteen days later, after being deprived of food for 15 h, blood was collected from the rat’s tail vein for glucose estimation. This value was used as the baseline blood glucose level. Then, Group 2 rats and Group 3 rats were given phenformin hydrochloride and 3-S once by intragastric administration. One hour later, rats of both the control and treated groups were injected intraperitoneally with glucose (2 g/kg body weight). Blood was collected from the rat’s tail vein at 30 min intervals up to 2 h [22] for glucose estimation using a glucometer.

#### 4.4.4. Determination of the Plasma Insulin Concentration

Kunming rats were deprived of food and allowed free access to water for 18 h. Then, diabetes was induced by the intraperitoneal injection of alloxan monohydrate at a dose of 50 mg/kg body weight. Seventy-two hours later, the rats were deprived of food for 4 h, and blood was collected from the rat’s tail vein for glucose level estimation. The rats with glucose levels above 10 mmol/L were randomly divided into 5 groups. The normal group had 10 normal rats. The normal group and the diabetic model control group were given an equivalent volume of saline by intragastric administration, once a day for 14 days. For the Group 3 animals, phenformin hydrochloride was administered at a dose of 25 mg/kg body weight by intragastric administration, once a day for 14 days. For Group 4, Group 5 and Group 6 animals, 3-S was given at a dose of 100 mg/kg, 300 mg/kg and 800 mg/kg body weight by intragastric administration, once a day for 14 days. On the fourteenth day, after being deprived of food for 12 h, Group 3, Group 4, Group 5 and Group 6 animals were given phenformin hydrochloride and different doses of 3-S by intragastric administration. Two hours later, blood was collected from the rat’s eyeballs for glucose level determination. The fasting serum insulin levels were determined using an insulin radioimmunoassay kit [23].

### 4.5. Statistical Analysis

All of the data were expressed as mean ± standard deviation (SD) of three replicates and were analyzed statistically by one-way analysis of variance using SPSS version 10.0 software. The statistical significance between the means of the experimental and control studies was established by Student’s *t*-test. The results were considered to be significant if *p* < 0.05, *p* < 0.01 or *p* < 0.001.

## 5. Conclusions

The hypoglycemic activity of differently regioselective chitosan sulfates in alloxan-induced diabetic rats was researched in this paper. The conclusions are as follows.

- Differently regioselective chitosan sulfates exhibited hypoglycemic activities.
- Hypoglycemic activity of low molecular weight sulfate chitosan was obviously higher.
- 3-S exhibited significantly hypoglycemic activities in alloxan-induced diabetic rats.
- 3-S could regulate the glucose tolerance effect.
- 3-S could significantly increase the insulin levels in experimentally induced rats.
–OSO_3_^−^ at the C3-position of chitosan is a key active site.

## References

[1] Triplitt C.L., Reasner C.A., Isley W.L., DiPiro J.T., Talbert R.L., Yee G.C., Matzke G.R., Wells B.G., Posey L.M. (2005). Diabetes Mellitus. Pharmacotherapy: A Pathophysiologic Approach.

[2] Kameswararao B., Kesavulu M.M., Apparao C. (2003). Evaluation of antidiabetic effect of momordica cymbelaria fruit in alloxan-diabetic rats. Fitoterapia.

[3] Pepato M.T., Mori D.M., Baviera A.M., Harami J.B., Vendramini R.C., Brunetti I.L. (2005). Fruit of the Jambolan tree and experimental diabetes. J. Ethnopharmacol..

[4] Rosak C. (2002). The pathophysiologic basis of efficacy and clinical experience with the new oral antidiabetic agents. J. Diabetes Complicat..

[5] Rahman Q., Zaman K. (1989). Medicinal plants with hypoglycaemic activity. J. Ethnopharmacol..

[6] Shanmugam K.R., Mallikarjuna K., Nishanth K., Kuo C.H., Reddy K.S. (2011). Protective effect of dietary ginger on antioxidant enzymes and oxidative damage in experimental diabetic rat tissues. Food Chem..

[7] Suba V., Murugesan T., Arunachalam G., Mardal S.C., Sahu B.P. (2004). Anti-diabetic potential of barleria lupilina extract in rats. Phytomedicine.

[8] Bing L., Wanshun L., Baoqin H. (2007). Antidiabetic effects of Chitooligo-saccharides on pancreatic islet cells and streptozotocin induced diabetic rats. World J. Gastroenterol..

[9] Nishimura S.I., Kai H., Shimada K., Yoshida T., Tokura S., Kurita K. (1998). Regioselective syntheses of sulfated polysaccharides: Specific anti-HIV-1 activity of novel chitin sulfates. Carbohydr. Res..

[10] Rahimi R., Nikfar S., Larijani B., Abdollahi M. (2005). A review on the role of antioxidants in the management of diabeties and its complications. Biomed. Pharmacother..

[11] Rudge M.V.C., Damasceno D.C., Volpato G.T., Almeida F.C.G., Calderon I.M.P., Lemonica I.P. (2007). Effect of Ginkgo biloba on the reproductive outcome and oxidative stress biomarkers of streptozotocin-induced diabetic rats. Braz. J. Med. Biol. Res..

[12] Prasad K. (2000). Oxidative stress as a mechanism of diabetes in diabetic BB prone rats: Effect of secoisolariciresinol diglucoside (SDG). Mol. Cell. Biochem..

[13] Kamalakkannan N., Stanely Mainzen Prince P. (2006). Rutin improves the antioxidant status in streptozotocin-induced diabetic rat tissues. Mol. Cell. Biochem..

[14] Xing R.E., Yu H.H., Liu S., Zhang W.W., Zhang Q.B., Li P.C. (2005). Antioxidant activity of differently regioselective chitosan sulfates *in vitro*. Bioorganic Med. Chem..

[15] Jelodar G., Mohsen M., Shahram S. (2003). Effect of walnut leaf, coriander and pomegranate on blood glucose and his topathology of pancreas of alloxan-induced diabetic rats. Afr. J. Tradit. Complement. Altern. Med..

[16] Szkudelski T. (2001). The mechanism of alloxan and streptozotocin action in B cells of the rat pancreas. Physiol. Res..

[17] Hamden K., Carreau S., Lajmi S., Aloulou D., Kchaou D., Elfeki A. (2008). Protective effect of 17 β-estradiol on hyperglycemia, stress oxidant, liver dysfunction and histological changes induced by alloxan in male rat pancreas and liver. Steroids.

[18] Lazarow A., Lazarow A. (1964). Alloxan diabetes and mechanism of β-cell damage by chemical agents. Experimental Diabetes.

[19] Colca J.R., Kotagel N., Brooks C.L., Lacy P.E., Landt M., Mc Danield M.L. (1983). Alloxan inhibition of a Ca^2+^ and calmodulin-dependent protein kinase in pancreatic islets. J. Biol. Chem..

[20] Edem D.O. (2009). Hypoglycemic effects of ethanolic extract of Aligator pear seed (*Persea americana* Mill) in rats. Eur. J. Sci. Res..

[21] Funom M. Etiology and pathophysiology of diabetes mellitus. http://ezinearticles.com/?Etiology-and-Pathophysiology-of-Diabetes-Mellitus&id=4353837.

[22] Matteucci E., Giampietro O. (2008). Proposal open for discussion: Defining agreed diagnostic procedures in experimental diabetes research. J. Ethnopharmacol..

[23] Buccolo G., David M. (1973). Quantitative determination of serum triglycerides by use of enzyme. Clin. Chem..

